# Uncoupling Protein-1 (UCP1) in the Adult Horse: Correlations with Body Weight, Rectal Temperature and Lipid Profile

**DOI:** 10.3390/ani11061836

**Published:** 2021-06-20

**Authors:** Francesca Arfuso, Claudia Giannetto, Maria Francesca Panzera, Francesco Fazio, Giuseppe Piccione

**Affiliations:** 1 Department of Veterinary Sciences, University of Messina, Polo Universitario dell’Annunziata, 98168 Messina, Italy; claudia.giannetto1@unime.it (C.G.); ffazio@unime.it (F.F.); gpiccione@unime.it (G.P.); 2 Department of Biomedical, Dental, Morphological and Functional Images, University of Messina, Via Consolare Valeria, 98125 Messina, Italy; mariafrancesca.panzera@unime.it

**Keywords:** uncoupling protein-1, lipid profile, horse, lipoproteins, rectal temperature, body weight

## Abstract

**Simple Summary:**

Uncoupling protein-1 (UCP1) plays important roles in the energy balance and regulation of metabolism and in the body temperature regulation. In this survey the correlation among UCP1, body weight, rectal temperature and lipid profile was assessed in the adult horse. The findings gathered from the current survey showed that UCP1 values are not related with body weight and temperature in studied animals, but they seem to be linked to pathways involved in lipid and lipoprotein metabolism.

**Abstract:**

This study aimed to evaluate the possible relationship among UCP1, body weight, rectal temperature and lipid profile in the horse. Thirty clinically healthy Italian Saddle geldings (6–10 years old) were enrolled after the informed owners’ consent. All horses were blood sampled and their body weight and rectal temperatures were recorded. On the sera obtained after blood centrifugation the concentration of UCP1, total lipids, phospholipids, non-esterified fatty acids (NEFAs), triglycerides, total cholesterol, high density lipoproteins (HDLs), low density lipoproteins (LDLs) and very low density lipoprotein fraction (VLDLs) was evaluated. Pearson’s correlation analysis was applied to assess the possible relationship between serum UCP1 concentration and the values of body weight, rectal temperature and lipid parameters. Serum UCP1 concentration showed no correlation with body weight, rectal temperature, HDLs and LDLs values, whereas it correlated negatively with serum total lipids, phospholipids, NEFAs, total cholesterol, triglycerides and VLDLs values (*p* < 0.0001). The findings suggest that in the adult horse the role of UCP1 is linked to the lipid metabolism rather than to thermoregulation.

## 1. Introduction

The thermogenin, also named uncoupling protein-1 (UCP1), is involved in energy balance, metabolism regulation and thermogenesis pathways [1,2]. This protein is mainly expressed in brown adipose tissue (BAT), the main site of adaptive thermogenesis known also as non-shivering heat production, in response to environmental temperature or diet [3,4].

Unlike the white adipose tissue (WAT), containing large lipid droplets and few mitochondria [2,4], the BAT, heavily innervated by sympathetic nerves, consists of fat cells with numerous mitochondria and small lipid droplets [2]. Although it had been established that BAT declines after puberty become rare in adults, nowadays the hypothesis that BAT can be found in adults has been consolidated [5]. Moreover, recently it has been demonstrated that BAT is both functionally and metabolically highly active in adults, especially after chronic exposure to cold [5]. Particularly, following proper stimulation, white adipocytes can acquire typical features of brown fat cells, including UCP1 expression [6], which may be used by the organism to increase metabolic energy expenditure. This process is termed “browning” of WAT and the implicated white adipocytes are indicated as beige fat cells which gain UCP1 expression ability and thermogenic potential [7,8,9,10,11]. Though the contribution of beige fat cells to systemic adaptive thermogenesis is under debate, the specific uncoupling of adipocyte mitochondria remains an attractive target for the development of anti-obesity drugs in humans and animals, and also to improve and renew the knowledge on the metabolic strategies implemented by organisms under certain conditions [1,12].

In the veterinary field, the investigation of UCP1 levels and their relationship with an animal’s metabolism has sparked a great interest, especially in livestock, under certain physiological conditions, such as growth or pregnancy, when metabolic pathways are constantly changing due to the metabolic adjustments needed for homeostasis maintenance. For instance, lower UCP1 levels are found in growing kids compared to adult goats [13], suggesting that this protein may serve to prevent the excessive energy loss in young animals. Also, as Arfuso et al., [14] described, UCP1 is involved in the lipid metabolism of periparturient mares, contributing to their metabolic adaptation.

Although data from experiments with UCP1 in reconstituted systems tend to indicate that fatty acids are essential for UCP1 function and that this protein increases lipolysis and fatty acid oxidation [15,16,17], there is a paucity of information regarding the relationship between the serum UCP1 levels and lipid profile in adult mammals including the horse.

To bring an additional contribution the aim of the current study was to assess whether there is a correlation between the UCP1 concentration and the values of body weight, rectal temperature and the various lipid components in the adult horse.

## 2. Materials and Methods 

### 2.1. Animals and Experimental Design. 

All the treatments, housing and animal care during the study were in accordance with the standards recommended by the European Directive 2010/63/EU for animal experiments. After the informed consent of the owners, 30 Italian Saddle geldings (6–10 years of age; mean body weight 449 ± 10 kg) were included in the study. All horses were managed similarly in the same place (a horse training center in Sicily, Italy, latitude 38°10′ 35′′ N; longitude 13°18′14′’ E), housed in individual boxes (3.5 × 3.5 m), subjected to the same environmental conditions (natural photoperiod, mean temperature 25 ± 4 °C, mean relative humidity 65 ± 5%).

The animals were fed twice per day (08.00 a.m.; 06.00 p.m.) with a total amount of approximately 2.5% of their body weight in dry matter intake (forage:concentrate ratio 70:30) and water was available ad libitum.

All the animals were clinically healthy (based on a thorough clinical examination) and free of internal and external parasites.

### 2.2. Sampling Procedures and Laboratory Analysis 

The same operator measured the body weight of each horse by means of a weighting platform (PS3000HD Heavy Duty Floor Scale, Breckwell, UK), the rectal temperature (RT) of the horses using a digital thermometer (HI92704, Hanna Instruments Bedfordshire, UK) inserted 15 cm in the rectum and collected blood samples in two 10 mL tubes with clot activator (Terumo Corporation, Tokyo, Japan) by jugular venipuncture at the same time, before the morning feeding of the animals. The tubes were transported to the laboratory in refrigerated bags.

The samples from the first tube (containing clot activator) were allowed to clot for 2 h at room temperature, then they were centrifuged at 1000× *g* for 20 min. The obtained sera were stored at −20 °C until analysis. These serum samples were analyzed to estimate the concentration of mitochondrial uncoupling protein-1 (UCP1) using an ELISA kit specific for equine species (Horse Uncoupling Protein-1, Mitochondrial (UCP1) ELISA Kit, Cat.No: MBS066225, MyBioSource, Inc. San Diego, California, USA) with a microwell plate reader (Sirio, SEAC, Florence, Italy). All calibrators and samples were run in duplicate and the samples exhibited parallel displacement to the standard curve for ELISA analysis. The sensitivity of this kit was 10 pg/mL and both the intra- and the inter-assay coefficients of variation for UCP1 were at <15%.

The samples from the second tube (containing clot activator) were centrifuged at 1300× *g* for 10 min, within 30 min from the collection, and the obtained sera were stored at −20 °C until analysis. The obtained samples were analyzed to estimate the concentration of total lipids, phospholipids, triglycerides, total cholesterol, high density lipoproteins (HDLs) and low density and lipoproteins (LDLs) using commercially available kits with an automated analyzer UV Spectrophotometer (model Slim SEAC, Florence, Italy). Very low density lipoprotein fraction (VLDLs) was estimated as one-fifth of the concentration of triglycerides [18].

### 2.3. Statistical analysis. 

All results were expressed as mean values ± standard deviation (±SD).

The normal distribution was proven by the Kolmogorov-Smirnov test performed on all data (*p* > 0.05). 

Pearson’s correlation coefficients were computed to evaluate the relationship between serum UCP1 concentration and the values of body weight, rectal temperature, serum lipid and lipoprotein indices in enrolled horses. A linear regression model (y = a + bx) was applied to determine the degree of correlation between these parameters. *p* values less than 0.05 were considered statistically significant. The statistical analysis was performed using the statistical software Prism v. 4.00 (Graphpad Software Ltd., San Diego, CA, USA, 2003).

## 3. Results

**Figure 1:**
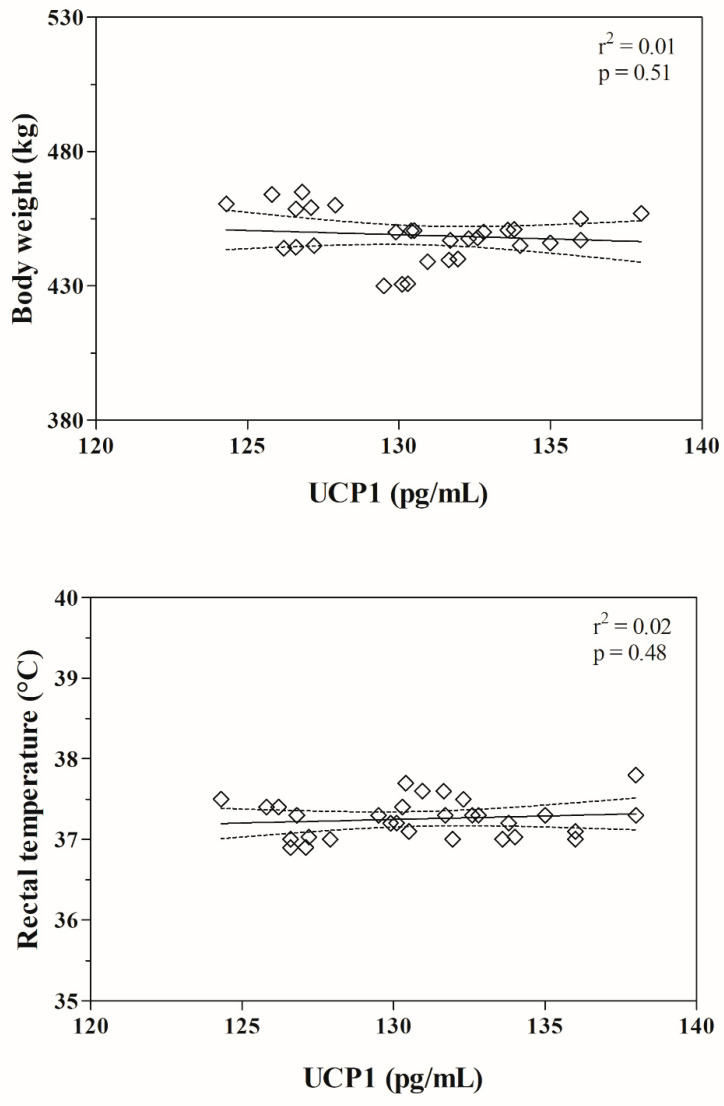
Results of linear regression model between the serum values of mitochondrial uncoupling protein-1 (UCP1) and the values of body weight and rectal temperature recorded in investigated horses.

**Figure 2:**
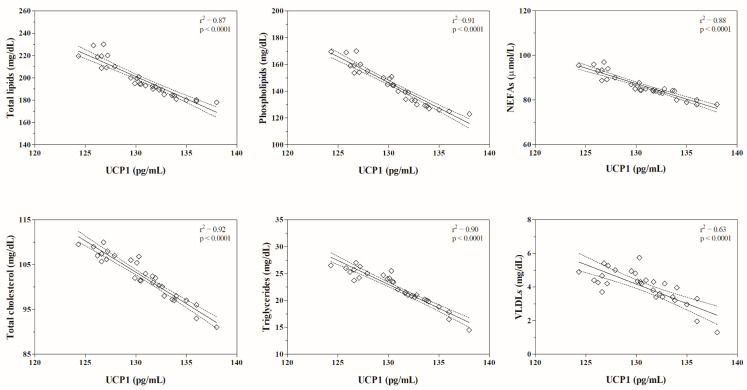
Results of linear regression model between the serum values of mitochondrial uncoupling protein-1 (UCP1) and the serum levels of lipid and lipoprotein indices obtained in investigated horses.

The serum UCP1 concentration did not correlate with body weight, rectal temperature, or HDLs and LDLs values, whereas it resulted negatively correlated with the serum values of total lipids, phospholipids, NEFAs, total cholesterol, triglycerides and VLDLs (Table 1). These findings were confirmed by the linear regression model results (Figure 1 and Figure 2).

## 4. Discussion

Although the role of UCP1 in regulation of metabolic and energy balance and in body temperature control is widely recognized [19], conflicting results on the presence of this protein in adult animals are shown by the scientific community. However, the recent discovery of the existence of the beige adipocytes showing both an expression panel partially typical of white adipocytes and a role in thermogenesis via expression of UCP1 [20,21,22], opened new insights in this field. The function, origin and localization of these fat cells are still not fully described and the literature currently available is limited to humans and mice.

According to the findings gathered in the current study, the UCP1 levels are not correlated with body weight values recorded in the investigated horses. In agreement with these results, a survey on genetically modified mice in which the UCP1 gene has been silenced (Ucp1−/− mice) suggested that the effect of UCP1 thermogenesis on body weight might be a consequence of the mechanisms carried out to body temperature maintenance [23].

The rectal temperature values recorded in the investigated horses lacked of correlation with the values of UCP1. Contrary to the findings herein observed, a statistically significant negative correlation between the values of rectal temperature and UCP1 was reported in a previous study carried out on adult goats and growing kids [13], where it has been hypothesized that, due to the UCP1 enrolment in the thermogenic process, higher levels of this protein would indicate a higher thermogenesis level leading to a body temperature rise. The lack of correlation between the values of UCP1 and rectal temperature found in the current study could suggest that other thermogenesis tools are involved in the regulation of body temperature in horses. Moreover, the environmental temperature conditions throughout the study (approximately 25 °C) were within the thermo-neutral zone, and this could have limited the expression of UCP1 and, consequently, the results regarding the relationship between UCP1 and body temperature.

Noteworthy, in our previous study carried out on horses, a negative relationship between UCP1 levels and the circadian clock gene Per2 involved in cold-induced adaptive thermogenesis has been found [24]. It has been supposed that this correlation is linked to the role of Per2 and UCP1 in the lipid metabolism, rather than to their role in thermoregulation. As a matter of fact, UCP1 is one of the targets involved in lipid metabolism within the peroxisome proliferator-activated receptor gamma (PPARγ). Per2 is the only clock gene protein able to interact with PPARγ, a master regulator of adipogenic differentiation and lipid metabolism [24].

In the current study UCP1 values displayed a significant correlation with serum total lipids, phospholipids, NEFAs, total cholesterol, triglycerides and VLDLs values, suggesting that thermogenin could be an integral component of cellular energy control, and that mechanisms of coordinated regulation may exist for UCP1 and other enzymes of oxidative metabolism in adult horses [19]. The correlation results were confirmed by a linear regression model applied on the data that highlighted a negative correlation between UCP1 concentration and the values of lipid parameters. The negative correlations found between the values of UCP1 and lipids and lipoproteins herein investigated could be explained by the fact that the main physiological activators of UCP1 are the fatty acids resulting from hormone-stimulated lipolysis [19,25]. Particularly, the reaction cascade activated by sympathetic nervous system terminals ends with the activation of protein kinase A leading to triacylglycerol lipase phosphorylation and activation that leads to the conversion of triacylglycerols into free fatty acids, which, ultimately, activate UCP1 [23]. An in vitro research, focused in the study of direct metabolic effects of forced UCP1 expression in the white adipocytes [22], reported a metabolic phenotype consistent with the energy dissipative function of UCP1, as suggested by previous reports [26,27,28,29,30], and by the current study. Indeed, a reduced accumulation of triglycerides has been observed and it has been hypothesized that the reduction in intracellular lipid by UCP1 expression could reflect a down-regulation of fat synthesis [22]. Although studies carried out in humans suggested that UCP1 gene polymorphisms are associated with the obesity pathogenesis and with the disequilibrium of lipid metabolism [26,27,28,29,30], these insights are not confirmed in other studies carried out on humans [31,32,33], as well as by the findings obtained in the current study, which showed no correlation between the values of UCP1 and the levels of HDLs and LDLs in investigated horses.

## 5. Conclusions

The findings gathered in the current study showed that UCP1 values are not correlated with body weight and temperature in adult horses, but they seem to be associated with lipid and lipoprotein metabolism pathways in adult horses. It might be hypothesized that thermogenin is an integral component of cellular energy control, and that mechanisms of coordinated regulation may exist for UCP1 and other enzymes of oxidative metabolism. Further investigations on this special but still little-known protein are worthy of interest in order to better clarify its functions and its possible involvement in metabolic/energetic adjustments that occur during the different life-phases of the animals and/or under stress conditions.

## Figures and Tables

**Table 1:** Results of correlation analysis (r Pearson; *p* value) between the levels of serum mitochondrial uncoupling protein-1 (UCP1) and the values of body weight, rectal temperature, total lipids, phospholipids, non-esterified fatty acids (NEFAs), total cholesterol, high density lipoproteins (HDLs), low density lipoproteins (LDLs) triglycerides and very low density lipoproteins (VLDLs) values calculated for horses enrolled in the study. *p* values < 0.05 were considered statistically significant.

**Parameters**	**Body Weight** **(kg)**				**Rectal Temperature** **(°C)**			
UCP1 (pg/mL)	r = −0.12 *p* = 0.51				r = 0.13 *p* = 0.48			
	**Total Lipids (mg/dL)**	**Phospholipids (mg/dL)**	**NEFAs** **(μmol/L)**	**Total Cholesterol (mg/dL)**	**HDLs (mg/dL)**	**LDLs (mg/dL)**	**Triglycerides (mg/dL)**	**VLDLs (mg/dL)**
UCP1 (pg/mL)	r = −0.93 *p* < 0.0001	r = −0.96 *p* < 0.0001	r = −0.94 *p* < 0.0001	r = −0.96 *p* < 0.0001	r = −0.11 *p* = 0.57	r = −0.47 *p* = 0.47	r = −0.95 *p* < 0.0001	r = −0.79 *p* < 0.0001

## Data Availability

The data presented in this study are available on request from the corresponding author.

## References

[1] Azzu V., Brand M. (2010). The on-off switches of the mitochondrial uncoupling proteins. Trends Biochem. Sci..

[2] Dalgaard L.T., Pedersen O. (2001). Uncoupling proteins: Functional characteristics and role in the pathogenesis of obesity and Type II diabetes. Diabetologia.

[3] Lowell B.B., Spiegelman B.M. (2000). Towards a molecular understanding of adaptive thermogenesis. Nature.

[4] Virtanen K., Nuutila P. (2011). Brown adipose tissue in humans. Curr. Opin. Lipidol..

[5] Virtanen K.A., Lidell M.E., Orava J., Heglind M., Westergren R., Niemi T., Taittonen M., Laine J., Savisto N.-J., Enerbäck S. (2009). Functional Brown Adipose Tissue in Healthy Adults. N. Engl. J. Med..

[6] Bouillaud F., Ricquier D., Tiraby C., Tavernier G., Lefort C., Larrouy D., Langin D. (2003). Acquirement of brown fat cell features by hu-man white adipocytes. J. Biol. Chem..

[7] Coskun T., Bina H.A., Schneider M.A., Dunbar J.D., Hu C.C., Chen Y., Moller D.E., Kharitonenkov A. (2008). Fibroblast growth factor 21 cor-rects obesity in mice. Endocrinology.

[8] Keipert S., Ost M., Johann K., Imber F., Jastroch M., Van Schothorst E.M., Keijer J., Klaus S. (2014). Skeletal muscle mitochondrial uncoupling drives endocrine cross-talk through the induction of FGF21 as a myokine. Am. J. Physiol. Metab..

[9] Fisher F.M., Kleiner S., Douris N., Fox E.C., Mepani R.J., Verdeguer F., Wu J., Kharitonenkov A., Flier J.S., Maratos-Flier E. (2012). FGF21 regulates PGC-1 and browning of white adipose tissues in adaptive thermogenesis. Genes Dev..

[10] Kharitonenkov A., Wroblewski V.J., Koester A., Chen Y.-F., Clutinger C.K., Tigno X.T., Hansen B.C., Shanafelt A.B., Etgen G.J. (2007). The Metabolic State of Diabetic Monkeys Is Regulated by Fibroblast Growth Factor-21. Endocrinology.

[11] Shabalina I.G., Petrovic N., de Jong J.M., Kalinovich A.V., Cannon B., Nedergaard J. (2013). UCP1 in brite/beige adi-pose tissue mitochondria is functionally thermogenic. Cell Rep..

[12] Keipert S., Jastroch M. (2014). Brite/beige fat and UCP1-is it thermogenesis?. Biochim. Biophys. Acta.

[13] Arfuso F., Rizzo M., Giannetto C., Giudice E., Fazio F., Piccione G. (2016). Age-related changes of serum mitochondrial uncoupling 1, rumen and rectal temperature in goats. J. Therm. Biol..

[14] Arfuso F., Giannetto C., Rizzo M., Fazio F., Giudice E., Piccione G. (2016). Serum levels of mitochondrial uncoupling protein 1, leptin, and lipids during late pregnancy and the early postpartum period in mares. Theriogenology.

[15] Schwartz M.W., Woods S.C., Porte D., Seeley R.J., Baskin D.G. (2000). Central nervous system control of food intake. Nature.

[16] Giacobino J.P. (2002). Uncoupling proteins, leptin, and obesity: An updated review. Ann. N. Y. Acad. Sci..

[17] Reidy S.P., Weber J.-M. (2002). Accelerated substrate cycling: A new energy-wasting role for leptin in vivo. Am. J. Physiol. Metab..

[18] Friedewald W.T., Levy R.I., Fredricson D.S. (1972). Estimation of low-density lipoprotein cholesterol in plasma, without use of the preparative ultracentrifuge. Clin. Chem..

[19] Brondani L.D.A., Assmann T.S., Duarte G.C.K., Gross J.L., Canani L.H., Crispim D. (2012). The role of the uncoupling protein 1 (UCP1) on the development of obesity and type 2 diabetes mellitus. Arq. Bras. Endocrinol. Metabol..

[20] Wu J., Boström P., Sparks L.M., Ye L., Choi J.H., Giang A.-H., Khandekar M., Virtanen K.A., Nuutila P., Schaart G. (2012). Beige Adipocytes Are a Distinct Type of Thermogenic Fat Cell in Mouse and Human. Cell.

[21] Petrovic N., Walden T.B., Shabalina I.G., Timmons J.A., Cannon B., Nedergaard J. (2010). Chronic peroxisome proliferator-activated receptor gamma (PPARgamma) activation of epididymally derived white adipocyte cultures reveals a population of thermogenically competent, UCP1-containing adipocytes molecularly distinct from classic brown adipocytes. J. Biol. Chem..

[22] Si Y., Palani S., Jayaraman A., Lee K. (2007). Effects of forced uncoupling protein 1 expression in 3T3-L1 cells on mitochondrial function and lipid metabolism. J. Lipid Res..

[23] Kozak L.P., Anunciado-Koza R. (2008). UCP1: Its involvement and utility in obesity. Int. J. Obes..

[24] Giannetto C., Arfuso F., Giudice E., Di Pietro S., Piccione G. (2021). Clock gene per 2 daily rhythm: Correlation with the serum level of uncoupling protein 1 (UCP1) in goat and horse. J. Therm. Biol..

[25] Arechaga I., Ledesma A., Rial E. (2001). The Mitochondrial Uncoupling Protein UCP1: A Gated Pore. IUBMB Life.

[26] Park M.J. (2005). Recent advances in regulating energy homeostasis and obesity. Korean J. Pediatr..

[27] Nagai N., Sakane N., Kotani K., Hamada T., Tsuzaki K., Moritani T. (2011). Uncoupling protein 1 gene −3826 A/G polymorphism is associated with weight loss on a short-term, controlled-energy diet in young women. Nutr. Res..

[28] Kim-Motoyama H., Yasuda K., Yamaguchi T., Yamada N., Katakura T., Shuldiner A.R., Akanuma Y., Ohashi Y., Yazaki Y., Kadowaki T. (1997). A mutation of the beta 3-adrenergic receptor is associated with visceral obesity but decreased serum triglyceride. Diabetologia.

[29] Esterbauer H., Oberkofler H., Liu Y.M., Breban D., Hell E., Krempler F., Patsch W. (1998). Uncoupling protein-1 mRNA expression in obese human subjects: The role of sequence variations at the uncoupling protein-1 gene locus. J. Lipid Res..

[30] Oh H.H., Kim K.S., Choi S.M., Yang H.S., Yoon Y. (2004). The effects of uncoupling protein-1 genotype on lipoprotein cholesterol lev-el in Korean obese subjects. Metabolism.

[31] Kotani K., Sakane N., Kurozawa Y., Kaetsu A., Okamoto M., Osaki Y., Kishimoto T. (2008). Polymorphism of Trp64Arg in beta3-adrenergic receptor gene and serum LDL-cholesterol concentrations in healthy Japanese. Ann. Clin. Biochem..

[32] Matsushita H., Kurabayashi T., Tomita M., Kato N., Tanaka K. (2003). Effects of uncoupling protein 1 and β3-adrenergic receptor gene polymorphism on body size and serum lipid concentrations in Japanese women. Maturitas.

[33] Schäffler A., Palitzsch K.D., Watzlawek E., Drobnik W., Schwer H., Schölmerich J., Schmitz G. (1999). Frequency and significance of the A→G (−3826) polymorphism in the promoter of the gene for uncoupling protein-1 with regard to metabolic parameters and adipocyte transcription factor binding in a large population-based Caucasian cohort. Eur. J. Clin. Investig..

